# Effects of Combined Application of Biogas Slurry and Chemical Fertilizer on Soil Aggregation and C/N Distribution in an Ultisol

**DOI:** 10.1371/journal.pone.0170491

**Published:** 2017-01-26

**Authors:** Xuebo Zheng, Jianbo Fan, Lei Xu, Jing Zhou

**Affiliations:** 1 Institute of Soil Science, Chinese Academy of Sciences, Nanjing, China; 2 University of Chinese Academy Sciences, Beijing, China; 3 National Engineering Research and Technology Center for Red Soil Improvement, Red Soil Ecological Experiment Station, Chinese Academy of Sciences, Yingtan, China; 4 Institute of Biology Resource, Jiangxi Academy of Sciences, Nanchang, China; RMIT University, AUSTRALIA

## Abstract

Unreasonable use of chemical fertilizer (CF) on agricultural soil leads to massive losses of soil organic carbon (SOC) and total nitrogen (TN) in tropical and subtropical areas, where soil conditions are unfavorable for aggregate formation. This study evaluated the effects of combined application of biogas slurry (BS) plus CF on soil aggregation and aggregate—associated C/N concentration and storage in an Ultisol. Six treatments included: no fertilizer (T1), CF only (T2), partial (15% (T3), 30% (T4) and 45% (T5)) substitution of TN with BS and BS only (T6). Soil mechanical—stable aggregates (MSAs) formation and stability as well as MSAs—associated C/N concentration and storage were observed in different aggregate sizes (>5, 5–2, 2–1, 1.0–0.5, 0.50–0.25 and <0.25 mm). The proportion of MSAs >5 mm significantly increased with BS substitution (T5), while the proportions of MSAs 1.0–0.5 mm, MSAs 0.50–0.25 mm and MSAs <0.25 mm significantly decreased. Both mean weight diameter and geometric mean diameter were highest in T5, which improved soil aggregation stability as well as resulted in significantly higher SOC and TN concentrations and storage in MSAs >0.5 mm that constituted 72–82% of MSAs. Stepwise regression analysis showed that MSAs >5 mm, SOC in MSAs >5 mm and TN in MSAs >5 mm were the dominant variables affecting aggregate stability. Meanwhile SOC in MSAs <0.25 mm and TN in MSAs 2–1 mm were independent variables affecting SOC and TN concentrations in bulk soils. Therefore, certain rate of combined application of BS plus CF is an effective, eco—friendly way to improve soil quality in an Ultisol.

## Introduction

Soil aggregate formation and stability play a major role in crop production and sustainable agricultural management [1]. The measurement of aggregate stability and dispersion is an indicator of soil structure [2]. Soil aggregation is the process by which aggregates of different sizes are joined and held together by different organic and inorganic materials [3], which is closely linked with the soil organic carbon (SOC) concentration. Organic carbon is an agent responsible for binding soil mineral particles together to create an aggregate hierarchy and it provides the flexible links between the external surfaces of clay domains [4]. It is also well known that SOC and total nitrogen (TN) are important for crop productivity as well as the sustainability of agro—ecosystems [5,6].

A good soil structure is critical for sustaining long—term crop production on arable soils for its influences on water status, workability, resistance to erosion, nutrient availability and plant growth [1]. However, intensive cultivation promotes soil degradation and significant losses of organic matter, leading to increased production costs (to maintain productivity) and higher CO_2_ emissions [7,8]. Degradation of soil structure occurs mostly as a result of the decrease in soil organic matter caused by low organic C input and excessive soil cultivation [9].

Organic materials are important soil amendments that sustain the productivity of soils in tropical and subtropical regions where there is low SOC content and low input of organic materials [10]. The addition of organic materials to fields can increase the SOC content by 50–150 kg/ha [8]. Use of organic materials has gained increasing importance within the last few years for sustainable agriculture and preventing soil degradation [1]. In agricultural management systems, recycled biogas slurry (BS) is a liquid by—product of anaerobic digestion of organic waste, such as crop straw, animal waste, municipal waste [11]. BS plays an important role in reducing intensive use of chemical fertilizers, which leads to resource conservation (reduced consumption of fossil fuels and mineral resources), climate change mitigation and soil quality maintenance [12]. Also, BS contains substantial organic carbon and crop nutrients that are attractive as fertilizers to return nutrients to soil ecosystems [4]. The application of BS is an important mechanism contributing to soil fertility through reducing soil erosion, mediating air permeability, water infiltration and nutrient cycling [13].

In southern China, chemical fertilizers have been widely used since the 1980s and cover 2.04 million km^2^ of the tropical and subtropical areas of this region [10]. Soils in this region contain a high content of clay, iron and aluminum oxides as well as a low content of organic matter, which are unfavorable for soil aggregate formation. In these circumstances, the unreasonable use of chemical fertilizer on agricultural land has led to a massive loss of SOC and TN [14]. A number of studies under organically and conventionally managed soils and intensively cropped soils with maize (*Zea mays L*.)—wheat (*Triticum aestivum L*.)—cowpea (*Vigna unguiculata*), respectively, have shown that balanced fertilization with nitrogen (N), phosphorus (P) and potassium (K) plus manures increases SOC concentration compared with treatments only using chemical fertilizers [15,16]. However, the response of soil aggregation and aggregate—associated SOC and TN concentrations to combined application of BS plus chemical fertilizer (CF) has been poorly studied. To better understand the effects of BS—CF application on SOC/TN concentrations and storage, it is necessary to conduct research at the aggregate scale. This could only be achieved by a fractionation procedure that separates the soil into ecologically meaningful subunits of the soil structure [17].

The aim of this study was to determine the effects of BS—CF combined application compared to no CF or BS, CF only and BS only treatments, in terms of soil aggregate formation and stability as well as SOC/TN concentrations and storage in a red soil (Ultisol) in southern China. We also determined how the combined BS—CF application influenced the relationships between the soil structure stability and aggregates (and SOC/TN concentrations in aggregates) as well as between SOC and TN concentrations in bulk soils and aggregates.

## Materials and Methods

### Study area and biogas slurry collection

**Fig 1:**
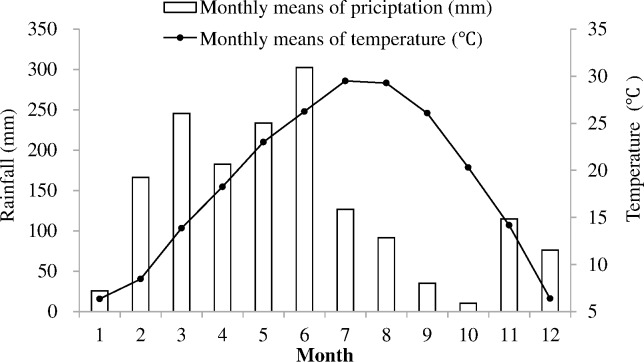
The average monthly rainfall (mm) and temperature (°C) in 2013–2014.

A field experiment was established in April 2013 at the Ecological Experimental Station of Red Soil, Chinese Academy of Sciences, which is located in Yujiang County, Jiangxi Province, southern China (28°12′N, 116.5°5′E), with a peanut (*Arachis hypogaea*)—fallow cropping rotation system. The climate is tropical and subtropical monsoon with mean annual precipitation of 1750 mm, mean annual evaporation of 1350 mm and mean annual temperature of 18°C. Rainfall during the experimental period (2013–2014) ranged between 10.3 and 302.2 mm and more than 80% of it fell during the monsoon months of February to June (Fig 1). The soil, derived from quaternary red clay, is classified as a Red Soil in the Soil Taxonomy System of China [18] and as an Ultisol in the Soil Taxonomy of the USDA [19]. In terms of soil texture at a depth of 0–20 cm, the soil contained 6.1% sand, 61.0% silt and 32.9% clay. The chemical properties of the field soil (0–20 cm) at the beginning of the experiment were as follows: pH 4.94 (soil/H_2_O = 1: 2.5), SOC 7.06 g/kg, TN 0.83 g/kg, NH_4_^+^—N 16.15 mg/kg and NO_3_^–^—N 3.90 mg/kg.

The BS was collected from a medium—sized pig farm with a biogas plant that included a well—mixed BS storage facility. The main feeds to the biogas plant reactor were a mixture of pig manure and urine. The operation temperature ranged between 35°C and 40°C and the minimum retention time was one year. The main characteristics of BS are shown in Table 1. Urea (with 46% N), calcium magnesium phosphate (with 12% P_2_O_5_) and potassium chloride (with 60% K_2_O) were selected as the N, P_2_O_5_, K_2_O sources, respectively.

**Table 1:** Main chemical properties of biogas slurry used in this study.

Parameters	Biogas slurry^a^
**pH**	7.59 ± 0.61
**EC (ds/m)**	21.9 ± 0.9
**DM (%)**	7.6 ± 1.2
**OC (mg/L)**	162 ± 17
**TN (mg/L)**	281 ± 9
**C: N**	0.58
**NH**_**4**_^**+**^**-N: TN**	0.96
**NH**_**4**_^**+**^**-N (mg/L)**	269 ± 8
**NO_3_^-^-N (mg/L)**	0.32 ± 0.03
**TP (mg/L)**	54 ± 4
**TK (mg/L)**	188 ± 11

^a^Values expressed as mean ± standard deviation (n = 3)

EC: electrical conductivity; DM: dry matter; OC: organic carbon; TN: total nitrogen; TP: total phosphorus; TK: total potassium.

### Experimental design

Six treatments were applied in which the BS was used as a substitute for CF as the source of TN, to varying degrees: T1, control (no CF or BS application); T2, CF only; T3, 15% BS—TN plus 85% CF—TN; T4, 30% BS—TN plus 70% CF—TN; T5, 45% BS—TN plus 55% CF—TN and T6, BS only. Thus, the quantities of BS applied in each treatment were calculated based on its TN concentration and the other macronutrients (P, K and additional N) were adjusted accordingly with CF. All treatments were arranged in a randomized complete block design with three replicates. Each plot measured 12.5 m by 4.8 m, and the distance between the different treatment blocks was 50 cm. Besides, the distances between lines and rows were 40 and 20 cm, respectively. For the CF and BS substitution treatments, the same final quantities of N, P_2_O_5_ and K_2_O were applied (N: P_2_O_5_: K_2_O, 120: 90: 135 kg/ha). The actual quantities of the BS applied were 6.41, 12.81, 19.22 and 42.73 × 10^4^ L ha^−1^, respectively, for T3, T4, T5 and T6 (Table 2). The organic carbon and nutrients added by the CF and BS within each treatment are shown in Table 2.

**Table 2:** Amounts of organic carbon and nutrients added by chemical fertilizers and biogas slurry.

Index	Chemical fertilizer						Biogas slurry					
	T1	T2	T3	T4	T5	T6	T1	T2	T3	T4	T5	T6
**BS (10**^**4**^**L/ha)**	/	/	/	/	/	/	/	/	6.41 (15%)	12.81 (30%)	19.22 (45%)	42.73 (100%)
**OC (kg/ha)**	/	/	/	/	/	/	/	/	10.38	20.75	31.13	69.19
**TN (kg/ha)**	/	120	102	84	66	/	/	/	18	36	54	120
**NH**_**4**_^**+**^**–N (kg/ha)**	/	/	/	/	/	/	/	/	17.23	34.46	51.88	114.87
**NO**_**3**_^**_**^**–N (kg/ha)**	/	/	/	/	/	/	/	/	0.02	0.04	0.06	0.13
**TP (P**_**2**_**O**_**5**_**) (kg/ha)**	/	90	84	78	73	52	/	/	6	12	17	38
**TK (K**_**2**_**O) (kg/ha)**	/	135	108	80	53	/	/	/	27	55	82	180

BS: biogas slurry; OC: organic carbon; TN: total nitrogen; TP: total phosphorus (P_2_O_5_); TK: total potassium (K_2_O). T1: control (no biogas slurry or chemical fertilizer; T2: chemical fertilizer (CF) only; T3: 15% biogas slurry (BS)–TN plus 85% CF–TN, T4, 30% BS–TN plus 70% CF–TN; T5: 45% BS–TN plus 55% CF–TN; and T6: BS only.

The appropriate quantities of BS for each treatment were manually spread onto the plowed soil (0–20 cm) by tillage using an open—head drum and ladle prior to sowing each year. After that, the supplementary CF (urea, calcium magnesium phosphate and potassium chloride) were surface—applied by hand and then incorporated into the soil when the soil was plowed for a second time before sowing. Thus, the BS and CF were used as basal fertilizers. Two peanut seeds were sown directly into each hole in early April 2013 and 2014, then the seedlings were thinned to ~140,000 plants ha^−1^ (equivalent to 840 plants per plot) in seedling stage and the mature peanuts were harvested in early August 2013 and 2014. The land was manually weeded at 15—day intervals during the peanut growing season. Pesticides were sprayed to control pests and diseases in all the plots when required. Soil sampling

Soils were sampled after the peanut harvest in early August 2014, from each plot at depth of 0–20 cm. Undisturbed soil samples with dimensions of 16 cm × 12 cm × 4 cm were obtained using a spade from three randomly selected sampling points in each plot for mechanical—stable aggregates (MSAs), SOC and TN concentrations measurement. Three randomly selected composite bulk soil samples were also collected from the same plots for SOC and TN concentrations measurement in bulk soils. Besides, three soil cores were taken randomly in each plot using a cutting ring to measure soil bulk density.

### Analytical methods

Undisturbed soil samples were air dried at room temperature. After that, all visible roots and crop residues were removed before breaking up the soil clods along their natural plane of weakness (<10 mm). MSAs were separated by dry—sieving air dried soils through a series of five sieves to isolate six aggregate size fractions. Air dried soils (1000 g) were evenly spread on the top of a 5 mm sieve, which was stacked on the top of a sequence of sieves with mesh sizes 2, 1, 0.5 and 0.25 mm, spaced about 5 cm apart vertically. The soil was immediately sieved to separate the MSAs for 5 min in a rotary sieve machine with a 75 stroke frequency [20]. The aggregates remained on the top 5 mm sieve were >5 mm MSAs and sieving procedure was for the 5–2 mm size with the next 2 mm sieve. The sieving procedure was then repeated for the other sieve sizes (1, 0.5 and 0.25 mm). Finally, MSAs <0.25 mm were collected from the stainless steel bottom. All MSAs were collected, weighed and stored for SOC and TN analysis. The aggregate fraction in each sieve represented the MSAs according to size classes: >5 mm (MSAs >5 mm), 5–2 mm (MSAs 5–2 mm), 2–1 mm (MSAs 2–1 mm), 1.0–0.5 mm (MSAs 1.0–0.5 mm), 0.50–0.25 mm (MSAs 0.50–0.25 mm), <0.25 mm (MSAs < 0.25 mm). Mean weight diameter (MWD) and geometric mean diameter (GMD) were calculated for each treatment using Kemper’s formula [21] as follows:

$$\text{MWD}=\sum_{\text{i}=1}^{\text{n}}\;Xi\times Wi$$ (1)

$$GMD=Exp\;[\frac{\sum_{i=1}^{n}\;WilnXi}{\sum_{i=1}^{n}\;Wi}]$$ (2)

where MWD and GMD are the mean weight diameter and geometric mean diameter, respectively, of MSAs (millimeters), X*i* is the mean diameter (mm) of the sieve size class (7.5, 2.5, 1.5, 0.75, 0.375 and 0.125 mm), W*i* is the weight of soil (g) retained on each sieve and n is the number of fractions (>5 mm, 5–2 mm, 2–1 mm, 1.0–0.5 mm and 0.50–0.25 mm).

Composite bulk soil samples were also air dried at room temperature, ground and passed through a 0.25 mm diameter sieve prior to the analysis of SOC and TN concentrations.

The following parameters were determined in the BS samples: EC and pH (directly, after sample homogenization) [22] and dry matter content, after drying to constant weight at 105 ^o^C. The organic carbon and TN were measured by automatic microanalysis (EuroVector elemental analyzer, Milan, Italy). After nitric acid—perchloric acid digestion, total P and K were analyzed by inductively coupled plasma—mass spectrometry [23]. NH_4_^+^ was measured through a modified colorimetric method, based on the Berthelot reaction [24]. NO_3_^−^ was determined by absorbance at 220 nm, and the values obtained were subtracted from the corresponding values at 275 nm, which were attributed to organic matter [25].

In soil, SOC in the bulk soils and MSAs was calculated from loss on ignition at 500^°^C for 24 h, then calculated by dividing the organic matter content by 1.72 [26]. TN in the bulk soils and MSAs was determined using the flow analyzer procedure after treatment with HClO_4_—H_2_SO_4_ [6]. Soil bulk density in the depth of 0–20 cm was determined on an oven—dry basis using the cylinder method [27].

### Calculations

The SOC and TN storage for different MSAs in topsoil (0–20 cm) were calculated as follows [28,29]:

SOC storage=SOC×BD×d×W×10 (3)

TN storage=TN×BD×d×W×10 (4)

Where SOC _storage_ and TN _storage_ refer to SOC and TN storage in each size class (10^4^ g/ha), SOC and TN refer to the SOC and TN concentrations of different size classes (g kg—1), W is the fraction weight of each aggregate size, BD is the soil bulk density (g/cm^3^), d is the depth of the soil profile (0.2 m) and the factor 10 is to adjust the units.

### Statistical analysis

All statistical analyses were carried out using SPSS (Statistical Package for Social Science) 13.0 (SPSS Inc., Chicago, IL, USA) for windows. Statistically significant differences were identified by one—way ANOVA and the least significant difference (LSD) at a significance level of *P* < 0.05 was used to identify differences between treatments means for MSAs, SOC/TN concentrations and storage. The relationships between GMD/MWD and the measured soil attributes were determined using stepwise regression analysis [6].

## Results

### Aggregate formation

**Fig 2:**
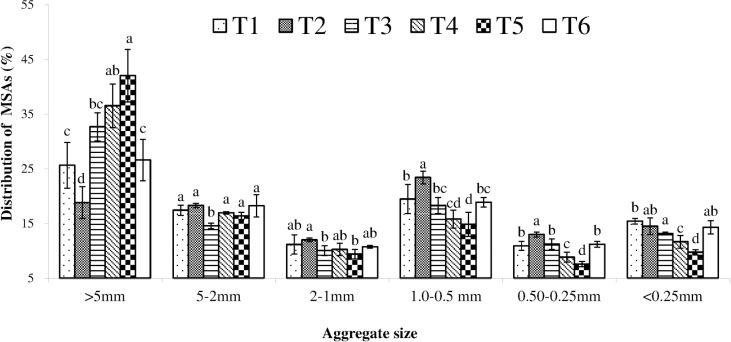
Distribution of MSAs under different treatments. Soil samples were collected at a depth of 0–20 cm after two years combined application of biogas slurry (BS) plus chemical fertilizer (CF). MSAs: mechanical—stable aggregates. T1: control (no BS or CF); T2: CF only; T3: 15% BS—TN plus 85% CF—TN, T4: 30% BS—TN plus 70% CF—TN; T5: 45% BS—TN plus 55% CF—TN; and T6: BS only. Values were mean (n = 3). vertical bars in the figures represent the standard deviation of mean (n = 3) and lower case letters indicate significant differences between treatments at the *P* < 0.05 level (LSD test). ANOVA source information is shown in S4 Table.

Significant differences in MSAs were observed under the different combined application of BS plus CF (Fig 2). MSAs >5 mm had a range of 18.82% in the T2 treatment to 42.02% in the T5 treatment and were significantly higher in the T5 treatment than in the other treatments except T4 (*P* <0.05). The MSAs >5 mm of T3 increased 7.04%, 13.87%, 6.09%, T4 increased 10.86%, 17.69%, 9.91% and T5 increased 16.37%, 23.20%, 15.42%, respectively, compared to treatments T1, T2 and T6. The highest proportion of MSAs 5–2 mm was observed in the T6 treatment compared with the other treatments (*P* > 0.05), whereas the lowest was found in the T2 treatment (*P* < 0.05). The MSAs 2–1 mm were in the range of 9.40% in the T5 treatment to 12.00% in the T2 treatment and was significantly higher in the T2 treatment compared with treatments T3 and T5 (*P* < 0.05). The lowest proportion of MSAs 1.0–0.5 mm, MSAs 0.50–0.25 mm and MSAs <0.25 mm was in the T5 treatment, which was significantly lower than that in the other treatments (*P* < 0.05).

### Aggregate stability and soil bulk density

The increase in MSAs >5 mm caused a significant increase in the MWD and GMD in treatment receiving combined application of BS plus CF compared with that observed in the other treatments (*P* < 0.05) (Table 3), especially in treatment T5, which was also significantly higher than treatments T3 and T4 (*P* < 0.05). No significant differences were observed among T1, T2 and T6 treatments for the GMD (Table 3). Further correlation analysis found that the relationship between MWD, GMD (x) and the percentage of aggregates >0.25 mm (y) conformed separately to the linear equations: y = 0.207x − 14.72 (R^2^ = 0.986, n = 18, *P* < 0.01) and y = 0.166x − 12.86 (R^2^ = 0.913, n = 18, *P* < 0.01).

**Table 3:** Soil properties included bulk density, porosity, MWD and GMD under different treatments.

Treatment	Bulk density^a^ (g/cm^3^)	Porosity (%)	SOC (g/kg)	TN (g/kg)	MWD(mm)	GMD(mm)
**T1**	1.29 ± 0.01 a	51.23 ± 0.15 d	7.35 ± 0.01 d	0.95 ± 0.02 d	2.91 ± 0.28 c	1.35 ± 0.11 cd
**T2**	1.20 ± 0.04 c	54.84 ± 1.00 b	8.30 ± 0.09 bc	1.03 ± 0.04 c	2.48 ± 0.21 d	1.18 ± 0.08 d
**T3**	1.17 ± 0.01 d	55.77 ± 0.28 a	8.72 ± 0.29 ab	1.11 ± 0.01 b	3.31 ± 0.17 bc	1.57 ± 0.11 c
**T4**	1.18 ± 0.00 cd	55.52 ± 0.09 ab	8.92 ± 0.08 a	1.14 ± 0.07 a	3.65 ± 0.27 ab	1.87 ± 0.19 b
**T5**	1.17 ± 0.00 d	55.89 ± 0.01 a	8.55 ± 0.62 ab	1.04 ± 0.05 c	4.02 ± 0.30 a	2.17 ± 0.16 a
**T6**	1.22 ± 0.01 b	53.79 ± 0.29 c	7.94 ± 0.05 c	1.04 ± 0.01 c	3.00 ± 0.21 c	1.42 ± 0.10 cd

^a^Soil samples were collected at a depth of 0–20 cm after two years combined application of biogas slurry (BS) plus chemical fertilizer (CF).

SOC: soil organic carbon; TN: total nitrogen; MWD: mean weight diameter; GMD: geometric mean diameter; Mean values ± standard deviation (n = 3) in the same row followed by the same letter were not significantly different using LSD test at *P* < 0.05 level. ANOVA source information is shown in S1 Table.

The combined application of BS plus CF caused a significant decrease in the soil bulk density with that observed in treatments T1, T2, T6, and especially treatment T5. In contrast to soil bulk density, treatment T5 had a highest soil porosity which was significant higher than T1, T2 and T6 treatments with an increase rate of 4.66%, 1.05% and 2.10%, respectively.

### Soils organic carbon in aggregate size fractions and bulk soils

The highest SOC concentration in bulk soils was observed in the T4 treatment, whereas the lowest was in the T1 treatment and was significantly higher in the T3, T4, T5 treatments than in the T1, T2 and T6 treatments (*P* < 0.05) (Table 3). But no significant differences were observed for SOC in bulk soils among T3, T4 and T5 treatments.

**Fig 3:**
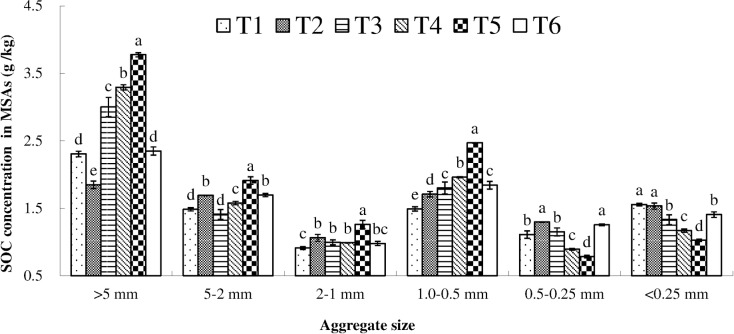
SOC concentration in MSAs under different treatments. Soil samples were collected at a depth of 0–20 cm after two years combined application of biogas slurry (BS) plus chemical fertilizer (CF). SOC: soil organic carbon (g/kg); MSAs: mechanical—stable aggregates. T1: control (no BS or CF); T2: CF only; T3: 15% BS—TN plus 85% CF—TN, T4: 30% BS—TN plus 70% CF—TN; T5: 45% BS—TN plus 55% CF—TN; and T6: BS only. Values were mean (n = 3). vertical bars in the figures represent the standard deviation of mean (n = 3) and lower case letters indicate significant differences between treatments at the *P* < 0.05 level (LSD test). ANOVA source information is shown in S5 Table.

The varying distribution of SOC concentrations in the MSAs depended on the application of fertilizer types (Fig 3). The highest SOC concentration in MSAs > 5 mm, MSAs 5–2 mm, MSAs 2–1 mm and MSAs 1.0–0.5 mm was all observed in treatment T5 and was significantly higher than that in the other treatments (*P* < 0.05). Adversely, the T5 treatment showed significantly lower SOC concentration in MSAs 0.50–0.25 mm and MSAs <0.25 mm compared with other treatments (*P* < 0.05).

At a soil depth of 0–20 cm, the SOC stock in MSAs > 5 mm, MSAs 5–2 mm, MSAs 2–1 mm and MSAs 1.0–0.5 mm significantly increased in treatment T5, while the SOC stock in MSAs 0.50–0.25 mm and MSAs <0.25 mm significantly decreased, compared with other treatments (*P* < 0.05, Table 4).

**Table 4:** SOC storage in MSAs under different treatments (10^4^ g/ha).

Treatment	Aggregate size (mm)					
	>5	5–2	2–1	1.0–0.5	0.50–0.25	<0.25
**T1**	152.49 ± 2.72 d	62.70 ± 1.01 d	22.08 ± 0.51 d	82.57 ± 1.80 c	31.20 ± 1.56 c	61.81 ± 0.81 a
**T2**	83.46 ± 2.51 e	70.56 ± 0.06 c	28.44 ± 1.34 b	64.79 ± 1.55 e	40.34 ± 0.04 a	53.32 ± 1.55 b
**T3**	229.53 ± 10.94 c	47.89 ± 2.48 e	23.39 ± 0.86 d	76.99 ± 3.77 d	30.18 ± 1.54 c	40.99 ± 2.23 d
**T4**	283.66 ± 3.38 b	63.04 ± 0.99 d	23.89 ± 0.01 cd	90.02 ± 0.34 b	18.57 ± 0.29 d	32.12 ± 0.66 e
**T5**	371.11 ± 3.07 a	81.75 ± 2.38 a	35.36 ± 1.71 a	135.20 ± 0.09 a	13.86 ± 0.34 e	23.46 ± 0.43 f
**T6**	152.37 ± 3.93 d	75.38 ± 1.11 b	25.61 ± 0.82 c	84.75 ± 2.63 c	34.49 ± 0.30 b	48.99 ± 1.46 c

MSAs: mechanical—stable aggregates; Mean values ± standard deviation (n = 3) in the same row followed by the same letter were not significantly different using LSD test at *P* < 0.05 level. ANOVA source information is shown in S2 Table.

### Total nitrogen in aggregate size fractions and bulk soils

Across all treatments, the highest TN concentration in bulk soils was observed in the T4 treatment, whereas the lowest was in the T1 treatment and was significantly higher in the T4 treatments than in the T1, T2, T3, T5 and T6 treatments (*P* < 0.05) (Table 3).

**Fig 4:**
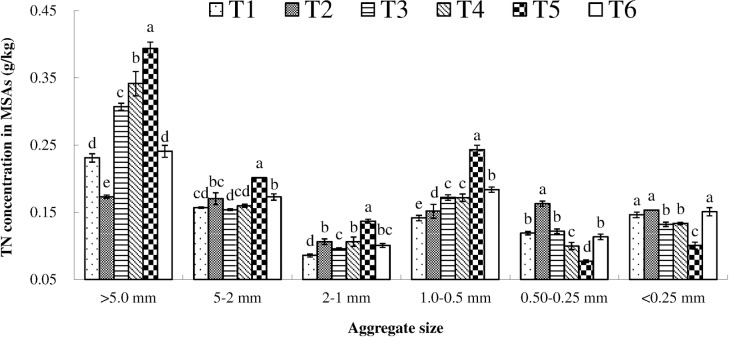
TN concentration in MSAs under different treatments. Soil samples were collected at a depth of 0–20 cm after two years combined application of biogas slurry (BS) plus chemical fertilizer (CF). TN, total nitrogen (g/kg); MSAs: mechanical—stable aggregates. T1: control (no BS or CF); T2: CF only; T3: 15% BS—TN plus 85% CF—TN, T4: 30% BS—TN plus 70% CF—TN; T5: 45% BS—TN plus 55% CF—TN; and T6: BS only. Values were mean (n = 3). vertical bars in the figures represent the standard deviation of mean (n = 3) and lower case letters indicate significant differences between treatments at the *P* < 0.05 level (LSD test). ANOVA source information is shown in S6 Table.

TN concentrations in MSAs varied after two years of combined application of BS plus CF (Fig 4). The highest TN concentration in MSAs > 5 mm, MSAs 5–2 mm, MSAs 2–1 mm and MSAs 1.0–0.5 mm were observed in treatment T5 and was significantly higher than that in the other treatments (*P* < 0.05). Adversely, the T5 treatment showed significantly lower TN concentrations in MSAs 0.50–0.25 mm and MSAs <0.25 mm compared with other treatments (*P* < 0.05).

Table 5 showed the variation in TN stock in MSAs at a soil depth of 0–20 cm after two years combined application of BS plus CF. TN storage in treatment T5 was significantly higher in MSAs > 5 mm, MSAs 5–2 mm, MSAs 2–1 mm and MSAs 1.0–0.5 mm, while significantly lower in MSAs 0.50–0.25 mm and MSAs <0.25 mm compared with other treatments (*P* < 0.05).

**Table 5:** TN storage in MSAs under different treatments (10^4^ g/ha).

Treatment	Aggregate size (mm)					
	>5	5–2	2–1	1.0–0.5	0.50–0.25	<0.25
**T1**	15.28 ± 0.41 d	6.63 ± 0.02 d	2.08 ± 0.07 d	7.84 ± 0.38 c	3.34 ± 0.07 b	5.81 ± 0.17 a
**T2**	7.82 ± 0.11 e	7.11 ± 0.04 c	2.85 ± 0.06 b	5.75 ± 0.21 e	5.07 ± 0.12 a	5.33 ± 0.01 b
**T3**	23.48 ± 0.39 c	5.23 ± 0.05 e	2.25 ± 0.04 d	7.35 ± 0.18 d	3.20 ± 0.09 bc	4.07 ± 0.10 c
**T4**	29.41 ± 1.55 b	6.39 ± 0.09 c	2.57 ± 0.17 c	7.88 ± 0.19 c	2.08 ± 0.11 d	3.67 ± 0.05 d
**T5**	38.70 ± 0.95 a	8.62 ± 0.38 a	3.84 ± 0.12 a	13.31 ± 0.56 a	1.37 ± 0.04 e	2.30 ± 0.12 e
**T6**	15.62 ± 0.57 d	7.68 ± 0.21 b	2.64 ± 0.07 c	8.45 ± 0.18 b	3.12 ± 0.11 c	5.26 ± 0.21 b

Mean values ± standard deviation (n = 3) in the same row followed by the same letter were not significantly different using LSD test at *P* < 0.05 level. ANOVA source information is shown in S3 Table.

### Relationship among measured soil attributes and parameters

Linear regression models between measured soil attributes and parameters, obtained by stepwise regression analysis, were shown in Table 6.

**Table 6:** Relationship among the measured soil attributes and parameters under different treatments.

	Regression model	R^2^
**Aggregate stability and MSAs**	Y_MWD_ = 0.073 MSA_>5 mm_ + 0.034 MSA_5–2 mm_ + 0.009 MSA_1.0–0.5 mm_ + 0.006 MSA_2–1 mm_ + 0.211;	0.999** (A)
	Y_GMD_ = 0.043 MSA_>5 mm_ + 0.292	0.956** (B)
**Aggregate stability and SOC**	Y_MWD_ = 0.765 SOC_MSA>5 mm_ + 1.116;	0.985** (C)
	Y_GMD_ = -1.148 SOC_MSA<0.25 mm_ -0.672 SOC_MSA0.50–0.25 mm_ + 3.850	0.991** (D)
**Aggregate stability and TN**	Y_MWD_ = 6.774 TN_MSA>5 mm_ + 1.332;	0.987** (E)
	Y_GMD_ = 4.387 TN_MSA>5 mm_ + 0.365	0.943** (F)
**SOC in bulk soils and MSAs**	Ysoc = -3.088 SOC_MSA>5 mm_-6.049 SOC_MSA5–2 mm_ + 4.336 SOC_MSA2–1 mm_-0.400 SOC_MSA0.50–0.25 mm_-12.623 SOC_MSA<0.25 mm_ + 39.469	0.965** (G)
**TN in bulk soils and MSAs**	Y_TN_ = 1.725 TN_MSA1.0–0.5 mm_-10.478 TN_MSA5–2 mm_ + 11.677 TN_MSA2–1 mm_-0.900 TN_MSA0.50–0.25 mm_ + 5.665 TN_MSA<0.25 mm_ + 0.592	0.976** (H)

R^2^: Correlation coefficient; Equation (A) Y_MWD_: MWD value; MSA _>5 mm_: MSAs >5 mm size fraction; MSA_5–2 mm_: MSAs 5–2 mm size fraction; MSA _2–1 mm_: MSAs 2–1 mm size fraction; MSA _1.0–0.5 mm_: MSAs 1.0–0.5 mm size fraction; Equation (B) Y_GMD_: GMD value; MSA _>5 mm_: MSAs >5 mm size fraction; Equation (C) SOC_MSA>5 mm_: SOC in MSAs >5 mm size fraction; Equation (D) SOC_MSA0.50–0.25 mm_: SOC in MSAs 0.50–0.25 mm size fraction; SOC_MSA<0.25 mm_: SOC in MSAs <0.25 mm size fraction; Equation (E)(F) TN_MSA>5 mm_: TN in MSAs >5 mm size fraction; Equation (G) SOC_MSA>5 mm_: SOC in MSAs >5 mm size fraction; SOC_MSA5–2 mm_: SOC in MSAs 5–2 mm size fraction; SOC_MSA2–1 mm_: SOC in MSAs 2–1 mm size fraction; SOC_MSA0.50–0.25 mm_: SOC in MSAs 0.50–0.25 mm size fraction; SOC_MSA<0.25 mm_: SOC in MSAs <0.25 mm size fraction; Equation (H) TN_MSA5–2 mm_: TN in MSAs 5–2 mm size fraction; TN_MSA2–1 mm_: TN in MSAs 2–1 mm size fraction; TN_MSA1.0–0.5 mm_: TN in MSAs 1.0–0.5 mm size fraction; TN_MSA0.50–0.25 mm_: TN in MSAs 0.50–0.25 mm size fraction; TN_MSA<0.25 mm_: TN in MSAs <0.25 mm size fraction.

**P < 0.01

The results indicated that the MWD was significantly and positively correlated to MSAs >5 mm, MSAs5–2 mm, MSAs 2–1 mm and MSAs 1.0–0.5 mm as shown in Eq.A (R^2^ = 0.999, *P* < 0.01), while the GMD was significantly and positively correlated to MSAs >5 mm as shown in Eq.(B) (R^2^ = 0.956, *P* < 0.01). This suggested that an increase in the macro—aggregates (>0.5 mm) resulted in improved aggregate stability, particularly MSAs >5 mm based on the discriminant coefficients in Eqs. (A) and (B).

Similarly, significant correlations were obtained between the MWD, GMD and SOC as well as TN concentrations in MSAs. The MWD was significantly and positively correlated to SOC concentrations in MSAs >5 mm (R^2^ = 0.985, *P* < 0.01) and to TN concentrations in MSAs >5 mm (R^2^ = 0.987, *P* < 0.01), as shown in Eqs. (C) and (E), respectively. The GMD was significantly and negatively correlated to SOC concentrations in MSAs 0.50–0.25 mm and MSAs <0.25 mm (R^2^ = 0.991, *P* < 0.01) and significantly and positively to TN concentrations in MSAs >5 mm (R^2^ = 0.943, *P* < 0.01), as shown in Eqs. (D) and (F), respectively.

The liner regression models of SOC and TN concentrations in bulk soils and MSAs were shown in Eqs. (G) and (H), respectively. Significant positive and negatively correlations were obtained between SOC concentrations in bulk soils and those in MSAs 2–1 mm and in MSAs >5 mm, MSAs 5–2 mm, MSAs 0.50–0.25 mm, MSAs <0.25 mm, respectively, as shown in Eq. (G) (R^2^ = 0.965, *P* < 0.01). A significant positive and negative correlations were obtained between TN in bulk soils and those in MSAs 2–1 mm, MSAs 1.0–0.5 mm, MSAs <0.25 mm and in MSAs 5–2 mm, MSAs 0.50–0.25 mm, respectively, as shown in Eq. (H) (R^2^ = 0.976, *P* < 0.01).

## Discussion

### Soil aggregate formation

Macro—aggregates (MSAs >5 mm) in treatment T5 which received combined application of BS plus CF was significantly higher than those in other treatments, accompanied by a significantly increase in SOC content. This suggested that a certain precentage (0.45: 0.55) of BS plus CF application based on TN content can lead to an increase in MSAs >5 mm as a result of transformation from 5–2, 2–1, 1.0–0.5, 0.50–0.25 and <0.25 mm aggregates size fraction. A positive relationship between SOC content and the percentage of aggregates >0.25 mm in an Utisol have been reported by Zheng et al. [30]. Therefore, the left of previous crop residues which contained abundant organic matter was the key to soil structural development due to the important role of organic matter played in soil aggregation [31]. Release of polysaccharides and organic acids during the decomposition of organic material contained in biogas slurry (Table 2) played a major role in formation of macro—aggregates [32]. The freshly added organic matter through BS functioned as nucleation sites for the growth of fungi and other soil microbes [33]. As consistent to our results, Angers et al. [34] found that manure application increased the formation of macro—aggregates (>1 mm) due to the accumulation of particulate organic carbon. Huang et al. [10] observed a significantly higher percentage of macro—aggregates and greater SOC concentrations in the bulk soils and >2 mm aggregate fraction after combined application of manure and chemical fertilizer. Iron—aluminum oxides and organic matter played important roles in the formation of micro—and macro—aggregates in red soils, respectively [35]. Tisdall et al. [36] speculated that macro—aggregates formed through the bonding of micro—aggregates by organic matter, while polysaccharides and ration bridging promoted micro—aggregate formation through bonding to soil particles. The biological and physicochemical properties of materials (especially metal ions, humic or fulvic acids and carbohydrate contents) played a role in the initial macro—aggregate formation [37,38]. Nutrition ions (e.g., NH_4_^+^, K^+^, Fe^2+^ and Mn^2+^) [39], humic acid and organic matter were abundant in BS [40], which ultimately contributed to the macro—aggregate formation, as consistent to the results of Alagöz et al. [1] who reported that large aggregates were obtained from smaller aggregates after humate application.

Moreover, particulate organic matter derived from organic matter decomposition served as substrate for microbial activity. Microbial biomass, especially fungal biomass, played a role in macro—aggregate formation [41] and fungal mycelium was a major factor in the transformation from micro—aggregates to macro—aggregates [41,42]. Previous study have revealed that combined application of BS plus CF increased the numbers of soil microorganisms (bacteria, fungi and actinomyces) [43], microbial biomass C and N and dehydrogenase and urease activities [22]. Thus, soil microbial growth could result in the production of microbial bonding materials that may also be responsible for macro—aggregate formation in this study. This was in agreement with the result of Six et al. [17] who found that soil fungi and other microorganisms utilized the more easily available C and produced mucilages which resulted in the formation of >0.25 mm aggregates.

### Aggregate stability and soil bulk density

Haynes and Swift [44] stressed that at least two significant phases were involved in aggregate formation: initial aggregation (driven by microbial polysaccharides) and stabilization (driven by humic materials). Table 3 showed higher MWD and GMD in soils treated with combined application of BS plus CF compared with no fertilizer and CF only treatments. This suggested that the combined application of BS plus CF, especially treatment T5, could promote aggregate stability in an Ultisol. Higher MWD and GMD values indicated the dominance of less erodible, larger aggregates in the soil [45] for positive relationships have been found between MWD, GMD and the percentage of macro—aggregates, respectively (Table 6). This was consistent with the results of Meng et al. [6] for manure application in salinized soil. The increase in aggregate stability due to the combined application of BS plus CF showed that they made an important contribution to the resistance of soil to external disruptive forces and to sustainable soil management. Furthermore, the stability of MSAs could be due to the bonding of SOC and TN contained in >5 mm aggregates (Table 6). There was a clear response of aggregate stability to the type of organic application. Yilmaz et al. [46] and Alagoz et al. [1] both showed the aggregate stability increased with organic applications, which may be linked to the input of persistent binding agents (e.g., humic acid and iron—aluminum oxides) through organic additives and macro—aggregate improvement. Piccool et al. [45] found that soil aggregate stability was improved and maintained with time more by the hydrophobic than by the hydrophilic components of organic matter.

Bulk density was inversely related to the changes in SOC [47]. The decrease in bulk density after the combined application of BS plus CF was the result of a dilution effect when the added organic material was mixed with the denser mineral fraction of the soil [47]. Adversely, total porosity increased after two years combined application of BS plus CF, accompanied with the increase of SOC content. This observed increase was caused by better aggregation of individual soil particles and a decrease of bulk density in the presence of high SOC content, which was consistent with Yang et al [48] who found that total soil porosity increased with the application of manure and mineral fertilizers.

### SOC/TN concentration and storage in aggregates

Continuous certain rate of combined application of BS plus CF (T5) caused a significant increase in SOC and TN concentration and stock in MSAs >5 mm (Tables 4 and 5; Figs 3 and 4). This increase was attributed mainly to high inputs of exogenous organic carbon (Table 2) from BS. Also, BS—CF application may increase SOC and TN content directly by promoting soil microbial activity [6] and indirectly increased crop productivity which resulted in more below—ground biomass (microbial and roots) [49]. All these effects would contribute to SOC and TN content increase in macro—aggregates after two years combined application of BS plus CF.

The stepwise regression analysis of MSAs and MWD, GMD indicated that MSAs >5 mm were the dominant independent variable affecting aggregate stability based on the discriminant coefficients in Eqs. (A) and (B). This suggested that aggregate stability was mainly related to MSAs >5 mm size fraction. The MWD was positively correlated with SOC concentration in MSAs >5 mm size fraction (*P* <0.01) as well as with TN concentration in MSAs >5 mm size fraction (*P* <0.01). Besides, the GMD was positively correlated with TN concentration in MSAs >5 mm size fraction (*P* <0.01), but negatively correlated with SOC concentrations in MSAs 0.5–0.25 mm and MSAs < 0.25 mm size fraction. This suggested that aggregate stability was significant positively correlated with SOC and TN concentrations in macro—aggregates (>5 mm), while significant negatively correlated with SOC concentrations in micro—aggregates (<0.5 mm). Meng et al. [6] and Rasool et al. [50] also reported that an increase in SOC concentration in macro—aggregates might be responsible for aggregate stability increasing. Nwadialo and Mbagwu [51] reported that SOC did not greatly affect micro—aggregate stability when the SOC concentrations were below a certain threshold. However, this result was inconsistent with Simansky et al [52] who reported a decrease of macro—aggregates which was followed a MWD decrease after N fertilizer application due to ammonium had a negative influence on soil structure [53].

The increase of SOC in macro—aggregates could help prevent aggregate breakdown so that increase the aggregate stability. Application of N fertilizer improved the SOC status, while organic carbon added resulted in significantly higher SOC and TN concentrations in both bulk soil and aggregates [54]. This study showed that N application in form of BS plus CF increased C accumulation in >0.5 mm aggregates size fraction, which with higher C added through substitution of N with BS. This suggested that good practices of BS—CF application could significantly enhance SOC and TN concentration in an Ultisol in southern China.

Moreover, the combined application of BS plus CF increased the SOC and TN stock associated with >0.5 mm aggregates compared to other treatments, which was consistent with Song et al [29] who found that the manure amendments increased the SOC stock with macro—aggregates. The higher SOC and TN storage in >0.5 mm aggregates in the BS—CF treatments indicated that SOC and TN content can be physically protected and reduced the turnover rate. Therefore, the BS—CF treatments not only increased the SOC and TN storage but also promoted the stable SOC and TN storage due to BS added.

## Conclusion

Combined application of BS plus CF on an Utisol significantly affected the distribution of MSAs and aggregate stability (MWD and GMD), as well as SOC and TN concentrations and storage in MSAs. In the Utisol, macro—aggregates (>5 mm) significantly accumulated in the soils with application of 45% BS—TN plus 55% CF—TN, as well as significantly increased the MWD and GMD compared with other treatments. SOC and TN concentrations in bulk soils and MSAs as well as their storage in >5 mm aggregates significantly increased in response to the combined application of BS plus CF. Stepwise regression analysis indicated that SOC in MSAs <0.25 mm and TN in MSAs 2–1 mm were the dominant independent variables affecting SOC and TN in bulk soils, respectively. Furthermore, MSAs >5 mm, SOC in MSAs >5 mm and TN in MSAs >5 mm were dominant independent variables affecting aggregate stability. However, SOC in MSAs <0.25 mm and MSAs 0.50–0.25 mm may have resulted in a decrease in aggregate stability. We concluded that certain rate (0.45: 0.55) of combined application of BS plus CF based on TN content can increase macro—aggregates (>5 mm) at the expense of 5–2, 2–1, 1.0–0.5, 0.5–0.25 and <0.25 mm aggregate size fractions, as well as increase aggregate stability, SOC and TN concentrations and storage in the macro—aggregates (>5 mm) and bulk soils. Therefore, the combined application of BS plus CF with a rate of 0.45: 0.55 based on TN content is a feasible practice for developing a healthy soil structure and improving the soil fertility. Simply stated, the BS—CF amendment is an effective, eco—friendly way of soil quality improvement in an Ultisol.

## Supporting Information

S1 Table ANOVA source information for Table 3. (PDF)

S2 Table ANOVA source information for Table 4. (PDF)

S3 Table ANOVA source information for Table 5. (PDF)

S4 Table ANOVA source information for Fig 2. (PDF)

S5 Table ANOVA source information for Fig 3. (PDF)

S6 Table ANOVA source information for Fig 4. (PDF)
