# Supplementary material for: Effects of Combined Application of Biogas Slurry and Chemical Fertilizer on Soil Aggregation and C/N Distribution in an Ultisol
Source: PLoS One. 2017 Jan 26;12(1):e0170491. doi: 10.1371/journal.pone.0170491 (PMC5268777; doi:10.1371/journal.pone.0170491)
Supplement: S1 Table — (PDF) [file pone.0170491.s001.pdf]

S1 Table ANOVA source information for Table 3

Bulk density (g/cm^3)	df	Sum of squares	Mean square	F value	p value
**Between Groups**	5	0.033	0.007	47.803	0.000
**Within Groups**	12	0.002	0.000		
**Total**	17	0.035			
**Porosity (%)**	**df**	**Sum of squares**	**Mean square**	**F value**	**p value**
**Between Groups**	5	47.584	9.517	47.803	0.000
**Within Groups**	12	2.389	0.199		
**Total**	17	49.973			
**SOC**	**df**	**Sum of squares**	**Mean square**	**F value**	**p value**
**Between Groups**	5	4.984	0.997	7.853	0.002
**Within Groups**	12	1.523	0.127		
**Total**	17	6.507			
**TN**	**df**	**Sum of squares**	**Mean square**	**F value**	**p value**
**Between Groups**	5	0.068	0.014	40.800	0.000
**Within Groups**	12	0.004	0.000		
**MWD**	17	0.072			
**0.5 - 0.25 mm**	**df**	**Sum of squares**	**Mean square**	**F value**	**p value**
**Between Groups**	5	4.574	0.915	15.315	0.000
**Within Groups**	12	0.717	0.060		
**GMD**	17	5.291			
**< 0.25 mm**	**df**	**Sum of squares**	**Mean square**	**F value**	**p value**
**Between Groups**	5	1.992	0.398	23.239	0.000
**Within Groups**	12	0.206	0.017		
**Total**	17	2.198			
