# Supplementary material for: Effects of Combined Application of Biogas Slurry and Chemical Fertilizer on Soil Aggregation and C/N Distribution in an Ultisol
Source: PLoS One. 2017 Jan 26;12(1):e0170491. doi: 10.1371/journal.pone.0170491 (PMC5268777; doi:10.1371/journal.pone.0170491)
Supplement: S2 Table — (PDF) [file pone.0170491.s002.pdf]

S2 Table ANOVA source information for Table 4

>5 mm	df	Sum of squares	Mean square	F value	p value
Between Groups	5	163130.363	32626.073	1153.625	0.000
Within Groups	12	339.376	28.281		
Total	17	163469.739			
**5 - 2 mm**	**df**	**Sum of squares**	**Mean square**	**F value**	**p value**
Between Groups	5	2099.500	419.900	167.444	0.000
Within Groups	12	30.093	2.508		
Total	17	2129.593			
**2 - 1 mm**	**df**	**Sum of squares**	**Mean square**	**F value**	**p value**
Between Groups	5	357.255	71.451	67.005	0.000
Within Groups	12	12.796	1.066		
Total	17	370.051			
**1.0 - 0.5 mm**	**df**	**Sum of squares**	**Mean square**	**F value**	**p value**
Between Groups	5	8775.192	1755.038	391.952	0.000
Within Groups	12	53.732	4.478		
Total	17	8828.924			
**0.5 - 0.25 mm**	**df**	**Sum of squares**	**Mean square**	**F value**	**p value**
Between Groups	5	1494.030	298.806	350.410	0.000
Within Groups	12	10.233	0.853		
Total	17	1504.262			
**< 0.25 mm**	**df**	**Sum of squares**	**Mean square**	**F value**	**p value**
Between Groups	5	2998.670	599.734	333.951	0.000
Within Groups	12	21.550	1.796		
Total	17	3020.220			
