# Supplementary material for: Effects of Combined Application of Biogas Slurry and Chemical Fertilizer on Soil Aggregation and C/N Distribution in an Ultisol
Source: PLoS One. 2017 Jan 26;12(1):e0170491. doi: 10.1371/journal.pone.0170491 (PMC5268777; doi:10.1371/journal.pone.0170491)
Supplement: S3 Table — (PDF) [file pone.0170491.s003.pdf]

S3 Table ANOVA source information for Table 5

>5 mm	df	Sum of squares	Mean square	F value	p value
Between Groups	5	1,867.460	373.492	562.237	0.000
Within Groups	12	7.972	0.664		
Total	17	1,875.432			
**5 - 2 mm**	**df**	**Sum of squares**	**Mean square**	**F value**	**p value**
Between Groups	5	20.202	4.040	121.806	0.000
Within Groups	12	0.398	0.033		
Total	17	20.600			
**2 - 1 mm**	**df**	**Sum of squares**	**Mean square**	**F value**	**p value**
Between Groups	5	5.760	1.152	120.089	0.000
Within Groups	12	0.115	0.010		
Total	17	5.875			
**1.0 - 0.5 mm**	**df**	**Sum of squares**	**Mean square**	**F value**	**p value**
Between Groups	5	98.514	19.703	195.917	0.000
Within Groups	12	1.207	0.101		
Total	17	99.721			
**0.5 - 0.25 mm**	**df**	**Sum of squares**	**Mean square**	**F value**	**p value**
Between Groups	5	23.911	4.782	531.694	0.000
Within Groups	12	0.108	0.009		
Total	17	24.019			
**< 0.25 mm**	**df**	**Sum of squares**	**Mean square**	**F value**	**p value**
Between Groups	5	25.933	5.187	313.498	0.000
Within Groups	12	0.199	0.017		
Total	17	26.132			
