# Supplementary material for: Effects of Combined Application of Biogas Slurry and Chemical Fertilizer on Soil Aggregation and C/N Distribution in an Ultisol
Source: PLoS One. 2017 Jan 26;12(1):e0170491. doi: 10.1371/journal.pone.0170491 (PMC5268777; doi:10.1371/journal.pone.0170491)
Supplement: S4 Table — (PDF) [file pone.0170491.s004.pdf]

S4 Table ANOVA source information for Fig 2

>5 mm	df	Sum of squares	Mean square	F value	p value
**Between Groups**	5	777.063	155.413	11.161	0.001
**Within Groups**	10	139.246	13.925		
**Total**	15	916.309			
**5 - 2 mm**	**df**	**Sum of squares**	**Mean square**	**F value**	**p value**
**Between Groups**	5	26.558	5.312	4.698	0.018
**Within Groups**	10	11.307	1.131		
**Total**	15	37.865			
**2 - 1 mm**	**df**	**Sum of squares**	**Mean square**	**F value**	**p value**
**Between Groups**	5	9.065	1.813	1.591	0.249
**Within Groups**	10	11.395	1.139		
**Total**	15	20.459			
**1.0 - 0.5 mm**	**df**	**Sum of squares**	**Mean square**	**F value**	**p value**
**Between Groups**	5	99.855	19.971	6.300	0.007
**Within Groups**	10	31.702	3.170		
**Total**	15	131.557			
**0.5 - 0.25 mm**	**df**	**Sum of squares**	**Mean square**	**F value**	**p value**
**Between Groups**	5	41.652	8.330	14.354	0.000
**Within Groups**	10	5.803	0.580		
**Total**	15	47.456			
**< 0.25 mm**	**df**	**Sum of squares**	**Mean square**	**F value**	**p value**
**Between Groups**	5	42.792	8.558	9.654	0.001
**Within Groups**	10	8.865	0.887		
**Total**	15	51.657			
