# Supplementary material for: Effects of Combined Application of Biogas Slurry and Chemical Fertilizer on Soil Aggregation and C/N Distribution in an Ultisol
Source: PLoS One. 2017 Jan 26;12(1):e0170491. doi: 10.1371/journal.pone.0170491 (PMC5268777; doi:10.1371/journal.pone.0170491)
Supplement: S5 Table — (PDF) [file pone.0170491.s005.pdf]

S5 Table ANOVA source information for Fig 3

>5 mm	df	Sum of squares	Mean square	F value	p value
**Between Groups**	5	7.738	1.548	295.681	0.000
**Within Groups**	12	0.063	0.005		
**Total**	17	7.801			
**5 - 2 mm**	**df**	**Sum of squares**	**Mean square**	**F value**	**p value**
**Between Groups**	5	0.478	0.096	56.128	0.000
**Within Groups**	12	0.020	0.002		
**Total**	17	0.498			
**2 - 1 mm**	**df**	**Sum of squares**	**Mean square**	**F value**	**p value**
**Between Groups**	5	0.221	0.044	29.581	0.000
**Within Groups**	12	0.018	0.001		
**Total**	17	0.239			
**1.0 - 0.5 mm**	**df**	**Sum of squares**	**Mean square**	**F value**	**p value**
**Between Groups**	5	1.626	0.325	141.390	0.000
**Within Groups**	12	0.028	0.002		
**Total**	17	1.653			
**0.5 - 0.25 mm**	**df**	**Sum of squares**	**Mean square**	**F value**	**p value**
**Between Groups**	5	0.619	0.124	102.747	0.000
**Within Groups**	12	0.014	0.001		
**Total**	17	0.633			
**< 0.25 mm**	**df**	**Sum of squares**	**Mean square**	**F value**	**p value**
**Between Groups**	5	0.651	0.130	75.852	0.000
**Within Groups**	12	0.021	0.002		
**Total**	17	0.672			
