# Supplementary material for: Effects of Combined Application of Biogas Slurry and Chemical Fertilizer on Soil Aggregation and C/N Distribution in an Ultisol
Source: PLoS One. 2017 Jan 26;12(1):e0170491. doi: 10.1371/journal.pone.0170491 (PMC5268777; doi:10.1371/journal.pone.0170491)
Supplement: S6 Table — (PDF) [file pone.0170491.s006.pdf]

S6 Table ANOVA source information for Fig 4

>5 mm	df	Sum of squares	Mean square	F value	p value
Between Groups	5	0.098	0.020	207.754	0.000
Within Groups	12	0.001	0.000		
Total	17	0.099			
5 - 2 mm	df	Sum of squares	Mean square	F value	p value
Between Groups	5	0.005	0.001	50.784	0.000
Within Groups	12	0.000	0.000		
Total	17	0.005			
2 - 1 mm	df	Sum of squares	Mean square	F value	p value
Between Groups	5	0.004	0.001	59.407	0.000
Within Groups	12	0.000	0.000		
Total	17	0.005			
1.0 - 0.5 mm	df	Sum of squares	Mean square	F value	p value
Between Groups	5	0.019	0.004	98.704	0.000
Within Groups	12	0.000	0.000		
Total	17	0.020			
0.5 - 0.25 mm	df	Sum of squares	Mean square	F value	p value
Between Groups	5	0.012	0.002	174.157	0.000
Within Groups	12	0.000	0.000		
Total	17	0.012			
< 0.25 mm	df	Sum of squares	Mean square	F value	p value
Between Groups	5	0.008	0.002	139.020	0.000
Within Groups	12	0.000	0.000		
Total	17	0.008			
